# Supplementary material for: Genome-Wide Identification and Analysis of the NPR1-Like Gene Family in Bread Wheat and Its Relatives
Source: Int J Mol Sci. 2019 Nov 27;20(23):5974. doi: 10.3390/ijms20235974 (PMC6928982; doi:10.3390/ijms20235974)
Supplement: Supplementary file 1 [file ijms-20-05974-s001.zip › ijms-606369 supplementary/Figures.docx]

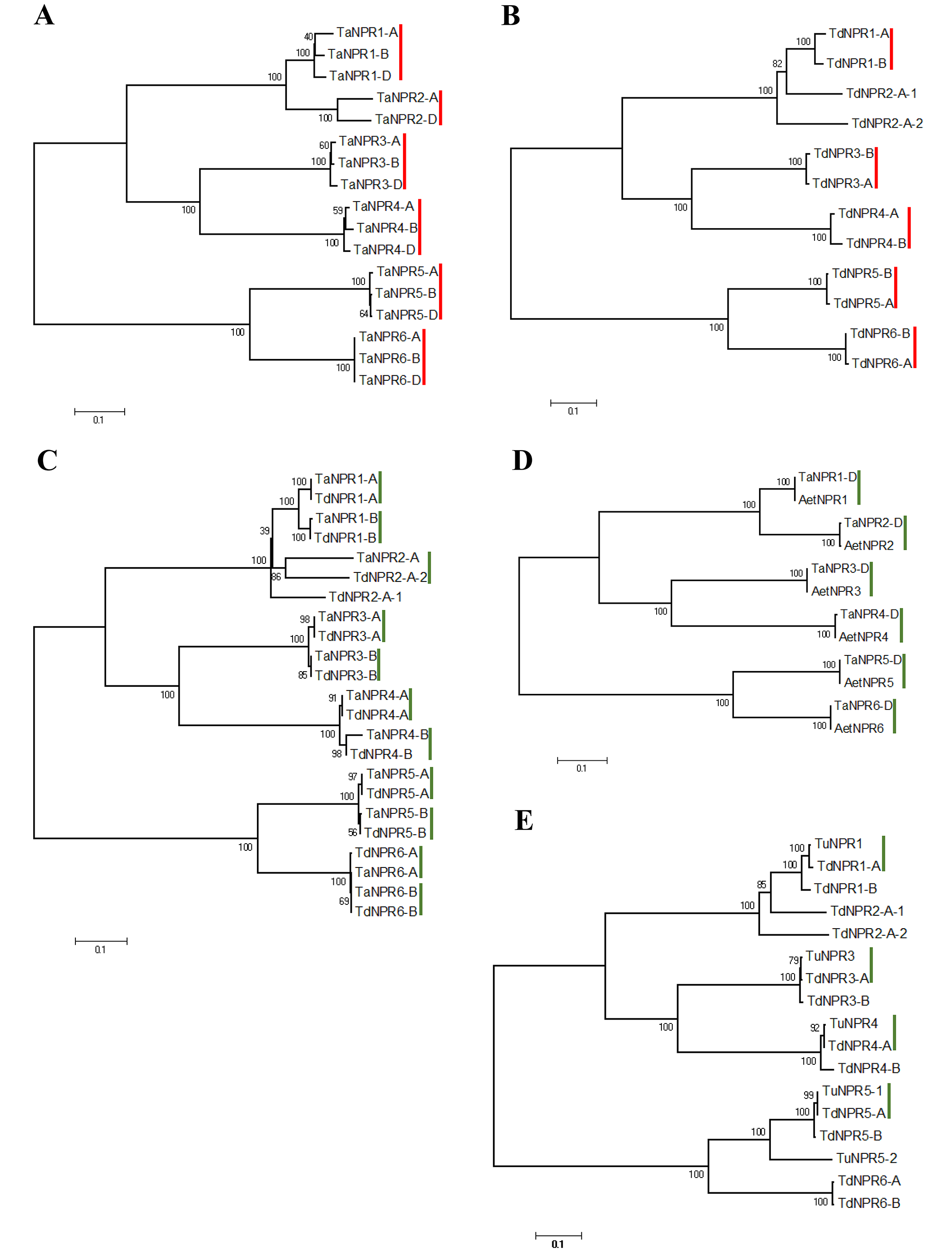


**Figure S1.** Analysis of *NPR1*-like orthologous and paralogous groups among bread wheat and its relatives. Phylogenetic trees for *NPR1*-like genes of *T. aestivum* **(A)**, *T. dicoccoides* **(B)***, T. aestivum* AB subgenome and *T. dicoccoides* **(C)**, *T. aestivum* D subgenome and *Ae. tauschii* **(D)**, and *T. dicoccoides* A subgenome and *T. uartu* **(E)** are constructed in MEGA v7.0 using the neighbor-joining (NJ) method with 1000 bootstrap replicates. The homeoalleles of one *NPR1*-like gene in the tetraploid and hexaploid wheat are marked by red lines. Orthologous gene pairs among bread wheat and its relatives are labeled by green lines.


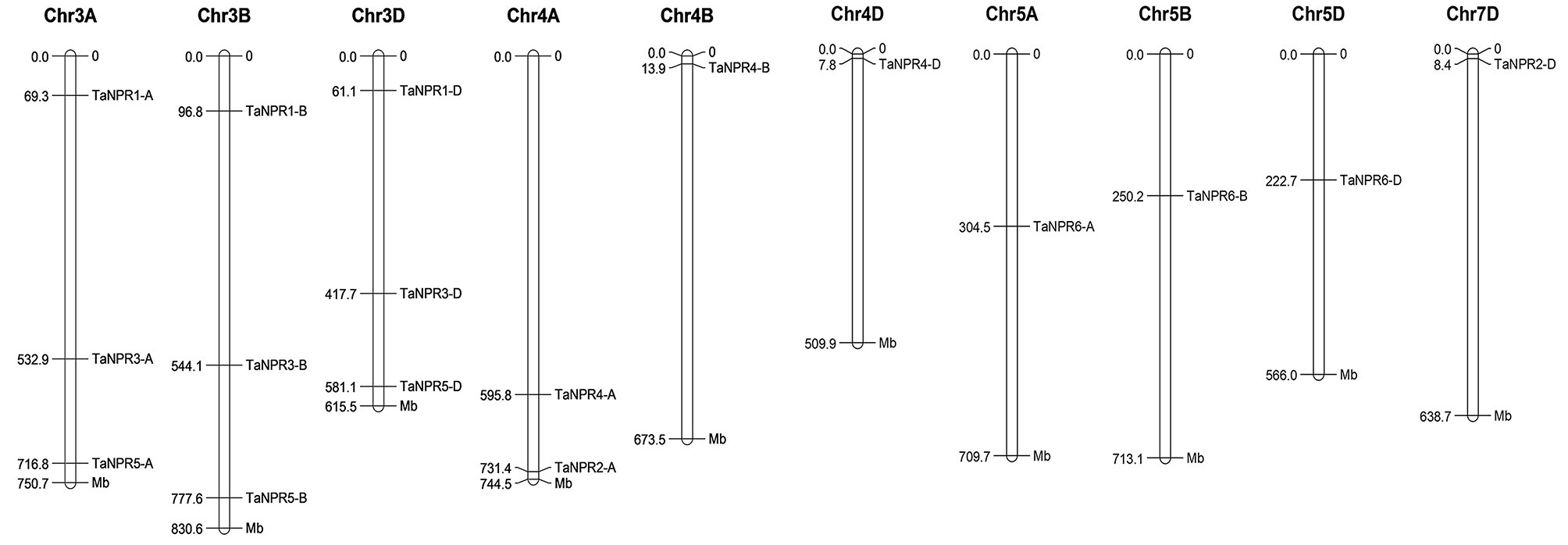


**Figure S2.** Physical location of *TaNPR1*-like genes on bread wheat chromosomes.
